# Novel aspects of the renin-angiotensin-aldosterone system in septic shock

**DOI:** 10.1016/j.cophys.2025.100894

**Published:** 2026-01-13

**Authors:** Christopher L Schaich, Ashish K Khanna, Mark C Chappell

**Affiliations:** 1 Hypertension & Vascular Research Center, Wake Forest University School of Medicine, Winston-Salem, NC, USA; 2 Department of Anesthesiology, Section on Critical Care Medicine, Atrium Health Wake Forest Baptist Medical Center, Winston-Salem, NC, USA; 3 Outcomes Research Consortium, Cleveland, OH, USA

## Abstract

Sepsis and septic shock are associated with high mortality rates and constitute the primary cause of death in intensive care units worldwide. Activation of the circulating renin-angiotensin-aldosterone system (RAAS) is an early event, and elements of the RAAS, including renin, Angiotensinogen, and ACE2, may be predictive of worse outcomes and higher mortality that reflect a failure to increase the circulating levels of the vasopressor Ang II. Emerging evidence suggests that dipeptidyl peptidase III (DPP3) is involved in the metabolism of Ang II, and higher DPP3 in septic shock may contribute to lower Ang II tone. The current review considers the role of a dysfunctional RAAS to maintain blood pressure and adequate tissue perfusion in septic shock.

## Introduction

The renin-angiotensin-aldosterone system (RAAS) is a critical endocrine system that supports adequate blood pressure and tissue perfusion [1–4]. Key components of this increasingly complex endocrine system include the protease renin, the precursor protein Angiotensinogen, and the peptidase Angiotensin Converting Enzyme (ACE) that contribute to the generation of the vasoactive peptide Angiotensin II (Ang II) (*Graphical Abstract*) [4]. Circulating Ang II binds primarily to the Angiotensin type 1 receptor (AT_1_R), stimulating various signaling pathways to increase blood volume and augment blood pressure [1]. Downstream effectors of the classical ACE-Ang II-AT_1_R axis include aldosterone, vasopressin, endothelin-1, and reactive oxygen species that contribute to increased vascular resistance and/or augment blood volume [2–4]. In turn, therapeutic approaches to block the RAAS, including ACE inhibitors and AT_1_R antagonists (ARBs), are first-line treatments for hypertension and other cardiovascular pathologies [4]. There is also compelling evidence for an alternative or nonclassical RAAS that primarily constitutes the ACE2-Ang-(1–7)-Mas receptor axis, which generally opposes the actions of Ang II [4–9]. Ang-(1–7) stimulates vasodilation through the release of nitric oxide (NO), promotes the renal excretion of Na+, and inhibits fibrotic and inflammatory events (*Graphical Abstract*) [4–7]. Indeed, both ACE inhibitors and ARBs increase the circulating levels of Ang-(1–7) through ACE2-dependent and non-dependent (neprilysin, prolyl oligopeptidase) pathways, which may contribute to their therapeutic actions [4–6].

A potentially new enzymatic component of the circulating RAAS is dipeptidyl peptidase III (DPP3), as emerging evidence suggests that DPP3 contributes to the regulation of the RAAS [9]. Preclinical studies show that DPP3 metabolized Ang II and influenced blood pressure in male but not female mice, suggesting marked sex differences in the function of the peptidase [10,11]. Administration of exogenous DPP3 increased renal blood flow in normotensive male mice that was associated with reduced circulating levels of Ang II [12]. Chronic treatment with DPP3 improved both cardiac and renal indices of injury in male diabetic mice (db/db) that may involve the metabolism of Ang II, given the protective effects of RAAS blockade in this type 2 diabetic model [13]. In contrast to ACE2 that releases Phenylalanine from the C-terminus of Ang II to generate Ang-(1–7), the aminopeptidase DPP3 cleaves the Aspartate-Arginine dipeptide from the N-terminus of Ang II to form Ang-(3–8) (Ang IV), which is rapidly hydrolyzed to Ang-(5–8) [10]. The greater rate of hydrolysis of Ang-(3–8) as compared to Ang II reflects the preference of DPP3 for smaller peptides of less than 10 amino acids. DPP3 is not specific for Ang II as DPP3 also hydrolyzed Ang-(1–7) to Ang-(3–7) and then to Ang-(5–7) [10,14]. Higher DPP3 activity in the cerebrospinal fluid (CSF) of sheep with perinatal glucocorticoid exposure inversely correlated with Ang-(1–7) CSF levels, suggesting a role for DPP3 in fetal programming events [5,15].

In critical illness such as septic shock, the pathways of the RAAS involved in the generation and metabolism of Ang II and Ang-(1–7) may be significantly altered to an extent that fails to support blood pressure and adequate tissue perfusion in these patients (*Graphical Abstract*). The current review evaluates this hypothesis regarding more novel aspects of dysregulation of the RAAS in septic shock, as well as the potential RAAS biomarkers renin, angiotensinogen, and DPP3.

### Regulation of the renin-angiotensin-aldosterone system

The release of renin from juxtaglomerular (JG) cells of the kidney is generally considered an early and committed event for the stimulation of the circulating RAAS [2–4]. Renin is stored within a distinct population of JG granules as prorenin, active renin, and a prorenin convertase, although the identity of this protease remains unknown. Stimulatory factors for the acute release of renin from JG cells include a reduction in blood pressure, sympathetic tone, succinate, and low tubular NaCl sensed by the macular densa (MD) cells that release prostaglandin E2, prostacyclin, and NO [16,17]. Factors that inhibit renin release include Ang II through JG AT_1_ receptors to reduce cAMP levels that constitute the short negative feedback loop, as well as adenosine, ANP, BNP, and apelin [16–18].

Although intact Angiotensinogen circulates at ‘saturating levels’ with respect to other RAAS components, the concentration of the precursor is at or below the Michaelis–Menten constant (Km) for renin, such that incremental changes in intact angiotensinogen may impact the generation of Ang I and subsequently Ang II [4,19,20]. Ang II, glucocorticoids, and various cytokines are known to stimulate the release of Angiotensinogen from the liver [2,4,19,20]. ACE and ACE2 are primarily membrane-bound peptidases that are likely not subject to acute regulation that occurs for renin or Angiotensinogen. Both enzymes are cleaved at the juxta-membrane stalk by other membrane-bound metalloproteinases termed ADAMs that may, in part, be activated by inflammatory events [4,5,21]. Shedding of ACE and ACE2 results in soluble forms that retain their full catalytic activity, which may increase circulating levels; however, shedding may deplete local tissue sites of these enzymes. In contrast to ACE and ACE2, DPP3 is primarily a soluble cytosolic peptidase (DPP3), and increased cytoplasmic DPP3 (cDPP3) in the circulation may reflect tissue damage and subsequent release of cDPP3; however, to our knowledge, the factors influencing the acute expression and/or release in cardiovascular tissues are not currently known.

### Renin-angiotensin-aldosterone system in septic shock

**Figure 1:**
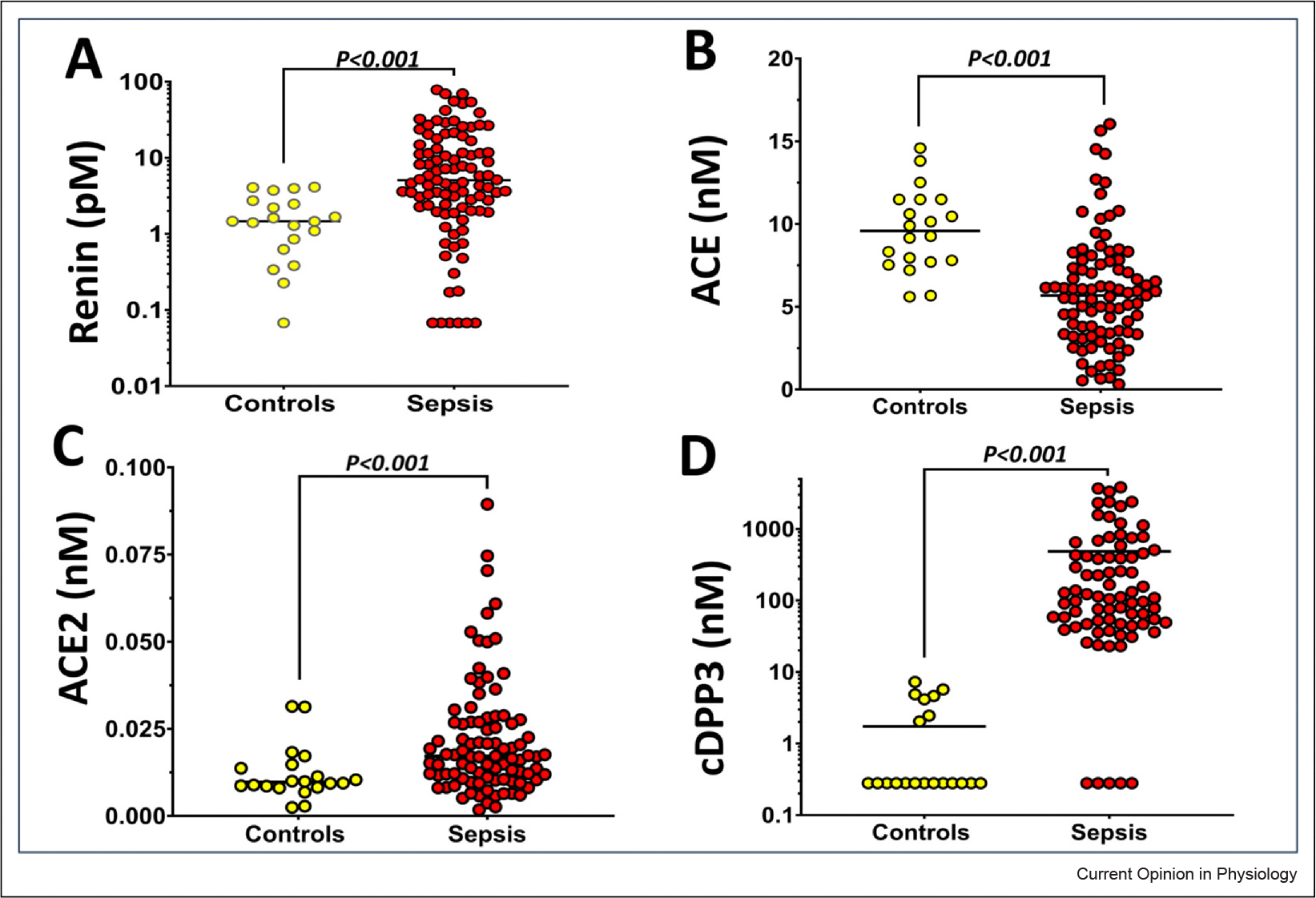
Lower ACE but higher Renin, ACE2, and cDPP3 concentrations in the circulation of sepsis patients. In comparison to the non-hospitalized control group (Control), sepsis patients (Sepsis) exhibited higher concentrations of active renin protein **(a)** but lower levels of ACE **(b)**. In contrast, sepsis patients exhibited higher concentrations of ACE2 **(c)** and cDPP3 **(d)** versus controls. Shown are scatter plots and the median value with Mann–Whitney analysis between groups. Renin and cDPP3 protein content were quantified by ELISA while ACE and ACE2 activities were converted to protein content [26]. Data adapted from Chappell et al. [26].

Acute activation of the RAAS is likely an early event in sepsis and septic shock to support overall blood pressure and tissue perfusion that reflects an increase in the circulating levels of renin. Reduced blood pressure and increased sympathetic activity are powerful stimulators of renin release from the kidney, which may be evident in septic shock patients. However, an attenuated response of other RAAS elements downstream from renin, particularly the ACE-Ang II-AT_1_R axis, may mitigate this attempt to maintain blood pressure and renal perfusion by stimulating renin release [7–9,22–27]. Moreover, increased ACE2 and cDPP3 may facilitate greater metabolism of Ang II that further contributes to an attenuated peptide response, also augmenting levels of Ang-(1–7) that functionally opposes Ang II (*Graphical Abstract*) [26,27]. Indeed, extensive profiling of the circulating RAAS components in sepsis patients from blood samples taken at hospital admission and prior to treatment revealed an overall increase in active renin content as measured by enzyme-linked immunosorbent assay but a marked decline (~40%) in ACE content as compared to a healthy, non-hospitalized cohort (Figure 1a,b). In contrast, both circulating cDPP3 and ACE2 concentrations were significantly higher in the sepsis group (Figure 1c,d), and the higher serum ACE to DPP3 ratio in control subjects was reversed in sepsis patients [26].

**Figure 2:**
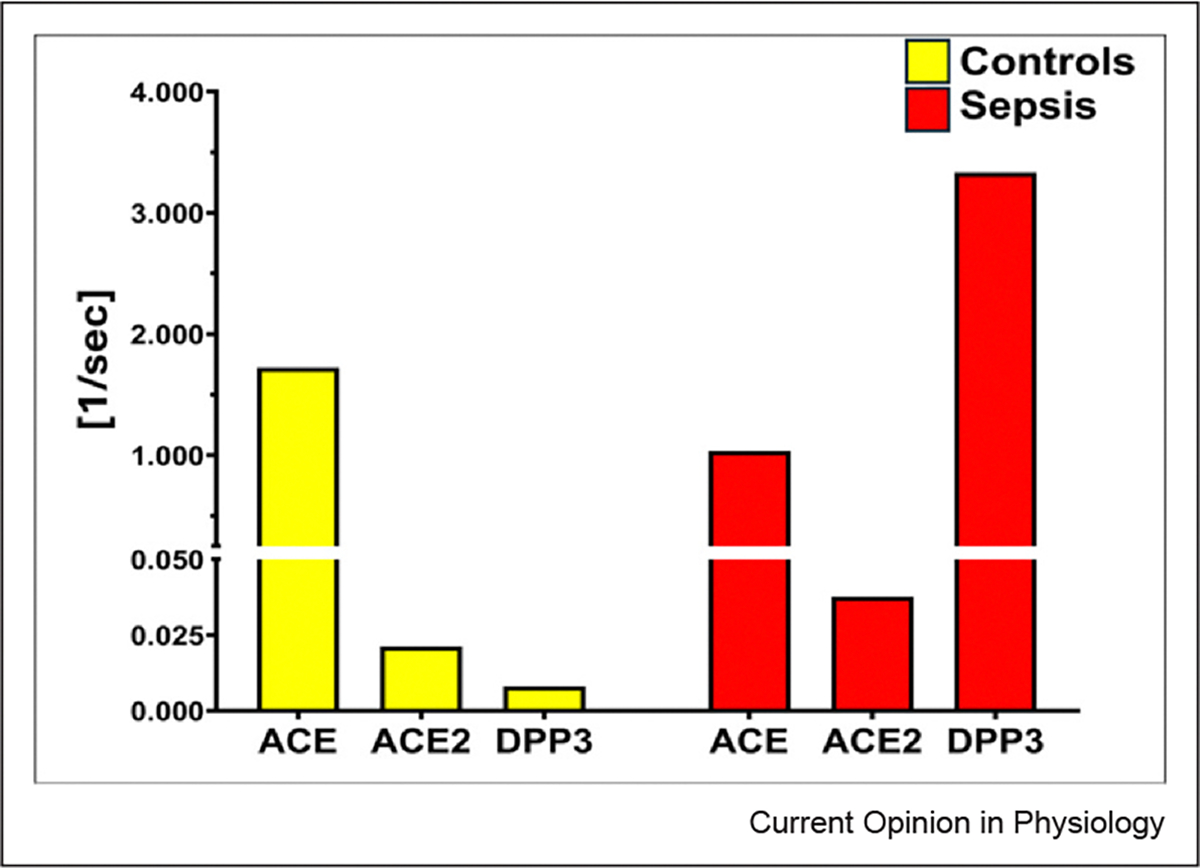
Comparison of the effective rate constants for ACE, ACE2, and cDPP3 in control and sepsis patients. Median enzyme concentration values (nM) were multiplied by their respective efficiency constants (Kcat/Km) for ACE/Ang I (1.8×10^−5^ M/s^−1^) [50], ACE2/Ang II (2.2×10^−6^ M/s) [50], and cDPP3/Ang II (3.0×10^−4^ M/s^1^) [10] to yield the effective rate constants. ACE was the predominant activity in the circulation of control subjects (Controls), while cDPP3 was the predominant activity in the circulation of sepsis patients (Sepsis). Data adapted from Chappell et al. [26].

Since these peptidases exhibit distinct efficiency constants [catalytic constant(kcat)/Km] for their respective substrates, we also compared the effective rate constants for ACE to generate Ang II, as well as cDPP3 and ACE2 to metabolize Ang II based on their median enzyme concentrations of the previous figure [(nM) × kcat/Km]. As shown in Figure 2, ACE was the dominant activity in the circulation of control subjects that would favor Ang II generation over the very low activities of ACE2 and cDPP3. In septic shock patients, cDPP3 was the predominant activity to contribute to the metabolism of Ang II, accompanied by lower ACE but still very low ACE2 activities (Figure 2).

**Figure 3:**
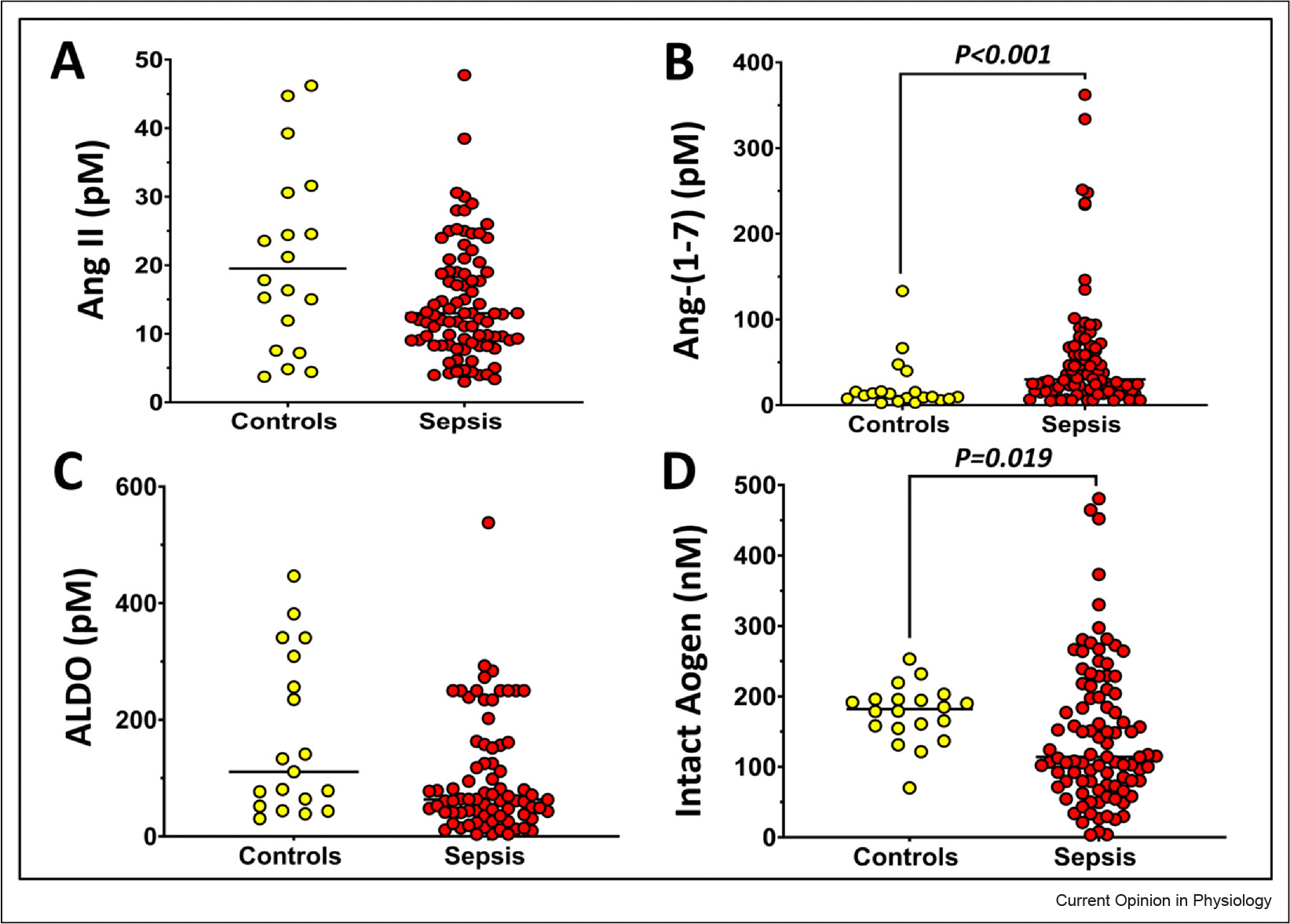
Comparison of circulating Ang II, Ang-(1–7), Aldosterone, and intact Angiotensinogen levels in control and sepsis patients. **(a)** Circulating Angiotensin II (Ang II) levels were not different between the control and sepsis patients. **(b)** Circulating Ang-(1–7) levels were significantly higher in the sepsis versus control subjects. **(c)** Circulating levels of Aldosterone (ALDO) were not different between the control and sepsis groups. **(d)** Circulating levels of intact Angiotensinogen (Aogen) containing the Ang I domain were lower in sepsis versus the control group. Shown are scatter plots and the median value with Mann–Whitney analysis between groups. Data adapted from Chappell et al. [26].

Although this comparison may not account for the membrane-anchored forms of ACE and ACE2 on those tissue surfaces in contact with circulating Angiotensin peptides, nor other peptidases capable of metabolizing Ang II, the circulating Ang II levels were not different between control and sepsis patients despite higher renin content (Figure 3a). Moreover, Ang-(1–7) levels were significantly higher in septic shock patients (Figure 3b), and the Ang II to Ang-(1–7) ratio was reduced in the sepsis group [26]. Also consistent with the lack of an Ang II response, circulating Aldosterone levels were not different between the control and sepsis groups (Figure 3c). Intact (containing the Ang I domain cleaved by renin) Angiotensinogen (Figure 3d), as well as total (both Ang I and des-Ang I domains) Angiotensinogen, were significantly lower in the sepsis versus the control group [26]. Overall, these data suggest that the inability to increase Ang II and Aldosterone in sepsis patients likely reflects multiple responses within the RAAS, including reduced levels of Angiotensinogen and ACE that culminate in the blunted generation of Ang II, as well as higher concentrations of cDPP3 and ACE2 that augment the metabolism of Ang II (*Graphical Abstract*). The increase in Ang-(1–7) may arise from greater processing of Ang II by ACE2, as well as reduced metabolism of Ang-(1–7) by lower ACE, although cDPP3 metabolism of Ang-(1–7) may also occur to limit an increase in Ang-(1–7). Garcia and colleagues [27] reported similar responses in the circulating RAAS, including elevated renin activity, higher Ang-(1–7) and ACE2, but reduced Ang II and ACE in septic shock patients using e*x vivo* processing conditions in serum to characterize the RAAS. The specific cellular mechanisms contributing to these events and their temporal sequence are currently unknown and await further study. In regard to DPP3, to our knowledge, Rehfeld et al. [28] first reported both markedly higher cDPP3 activity and enzyme concentration among hospitalized patients with severe sepsis and septic shock as compared to a healthy control cohort, although the status of other RAAS components was not evaluated. Preclinical studies find that cDPP3 inhibition by an antibody to the enzyme’s active site (Procizumab) was beneficial in septic shock [29,30] while a preliminary clinical study reported improved outcomes in patients with cardiogenic shock with Procizumab [31].

### Renin-angiotensin-aldosterone system biomarkers

Higher circulating levels of active renin (> 5 pM or 185 pg/mL) that are associated with poor clinical outcomes in septic shock patients [26,32] supports the renin response in severe sepsis and septic shock [33–36], catecholamine-resistant vasodilatory shock [37], and a heterogeneous group of critically ill patients [38]. In fact, Bellomo et al. [37] reported a comparable median renin value of 4.7 pM (174 pg/mL) by direct enzyme-linked immunosorbent assay in their patient cohort with vasodilatory shock, while Jeyaraju et al [35] find a baseline renin value of 4.3 pM (159 pg/mL) for in-hospital mortality of septic shock patients. Possibly reflecting the high renin content, we also reported that lower intact Angiotensinogen (< 115 nM or 5.8 μg/mL) outperformed renin and lactate as a predictor of higher mortality in sepsis patients that suggesting an inability to maintain or replenish circulating levels of Angiotensinogen may contribute to inappropriately low Ang II levels [39]. Reduced levels of intact Angiotensinogen in septic shock may also result in an underestimation of plasma renin activity, the more typical renin assay, which measures the generation of Ang I from endogenous Angiotensinogen.

As for DPP3, Rehfeld et al. [28] reported that plasma concentrations of the enzyme distinguished septic shock patients who survived at least 28 days and non-survivors. In a larger study, patients with severe sepsis or septic shock exhibited higher cDPP3 concentrations that were associated with a greater need for organ support and vasopressors upon hospital admission, longer duration of vasopressor support, mechanical ventilation or renal replacement therapy, higher need for fluid load, and greater 28-day mortality that outperformed lactate [40]. Conversely, lower cDPP3 concentrations (< 40 ng/mL or 482 pM) after 24 hours were associated with improved organ function and a lower 28-day mortality [40]. Higher DPP3 levels are also associated with organ dysfunction or death in cardiogenic shock [41–43], severe burn [44], severe COVID-19 [45,46], and in mixed intensive care unit populations [47,48]. Finally, Busse et al. [46] showed that in contrast to circulating renin and lactate, higher cDPP3 concentrations (≥41 ng/mL or 494 pM) associated with subsequent need for vasopressor support and fewer days free of vasopressor support in hospitalized COVID-19 patients without shock at baseline.

## Conclusions

Given the potential dysregulation of the RAAS in sepsis and septic shock, the accurate and rapid assessment of its components, including renin, intact Angiotensinogen, and cDPP3, may have potential prognostic value to guide treatment regarding Ang II replacement therapy or cDPP3 inhibition [22,23,32,37,49]. Indeed, treatment with exogenous Ang II or the DPP3 antibody Procizumab was recently shown to improve clinical outcomes in shock [31,37]. Further work is needed to determine the patient phenotype most likely to benefit from either Ang II treatment and/or cDPP3 blockade, as well as the optimal therapeutic window and potential interactions cDPP3 may have with Ang-(1–7) or other vasoactive peptides.

## Data Availability

Data will be made available on request.
